# Learning and processing of nonverbal symbolic information in bilinguals and monolinguals

**DOI:** 10.3389/fpsyg.2014.01147

**Published:** 2014-10-16

**Authors:** Henrike K. Blumenfeld, Ashley M. Adams

**Affiliations:** ^1^School of Speech, Language and Hearing Sciences, San Diego State UniversitySan Diego, CA, USA; ^2^Department of Speech and Hearing Science, Arizona State UniversityTempe, AZ, USA

**Keywords:** bilingualism, learning, nonlinguistic processing, priming, competition resolution, inhibition, auditory word identification, vocabulary

## Abstract

Bilinguals have been shown to outperform monolinguals on word learning and on inhibition tasks that require competition resolution. Yet the scope of such bilingual advantages remains underspecified. We compared bilinguals and monolinguals on nonverbal symbolic learning and on competition resolution while processing newly-learned material. Participants were trained on 12 tone-to-symbol mappings, combining timbre, pitch, and duration of tones. During subsequent processing, participants viewed a display with four symbols, and were instructed to identify the symbol that matched a simultaneously-presented tone. On competition trials, two symbols matched the tone in timbre and pitch, but only one matched the tone on timbre, pitch, and duration. No learning differences emerged between 27 Spanish-English bilinguals and 27 English monolinguals, and more successful learners performed better on the Peabody Picture Vocabulary task. During the processing task, competition trials yielded responses with lower accuracies and longer latencies than control trials. Further, in both groups, more successful learning of tone-to-symbol mappings was associated with more successful retrieval during processing. In monolinguals, English receptive vocabulary scores also influenced retrieval efficiency during processing, with English/Spanish vocabulary less related to the novel processing task in bilinguals. Finally, to examine inhibition of competing stimuli, priming probes were presented after each tone-symbol processing trial. These probes suggested that bilinguals, and to a lesser extent monolinguals, showed residual inhibition of competitors at 200 ms post-target identification. Together, findings suggest that learning of novel symbolic information may depend in part on previous linguistic knowledge (not bilingualism *per se*), and that, during processing of newly-learned material, subtle differences in retrieval and competition resolution may emerge between bilinguals and monolinguals.

## Introduction

A growing literature suggests that bilinguals may at times outperform monolinguals on inhibitory control (e.g., Bialystok et al., 2004; Martin-Rhee and Bialystok, 2008). Bilinguals may also be better word learners than monolinguals (e.g., Kaushanskaya and Marian, 2009). However, bilingual advantages over monolingual peers are not always identified in learning (e.g., Kaushanskaya and Rechtzigel, 2012) or processing tasks (e.g., Hilchey and Klein, 2011; Blumenfeld and Marian, 2014; Paap and Yunyun, 2014), perhaps in part because younger monolinguals and bilinguals, who are at their cognitive peak, may perform near ceiling, potentially obscuring more subtle group differences (e.g., Bialystok et al., 2008). It is also likely that bilingual cognitive advantages are constrained by numerous factors, including participants' age (e.g., Bialystok et al., 2005, 2008; Bialystok, 2006; Salvatierra and Rosselli, 2010), proficiency (Costa et al., 2008; Vega and Fernandez, 2011; Singh and Mishra, 2012; Blumenfeld and Marian, 2013), years of functional bilingualism (Luk et al., 2011), daily language immersion (e.g., Tao et al., 2011), and the nature of the task (e.g., Bunge et al., 2002; Bialystok and Senman, 2004; Blumenfeld and Marian, 2014). Where bilingual advantages do arise, it has been proposed that this is due to recruitment of cognitive mechanisms to support bilingual processing demands (e.g., Kroll, 2008). Such an explanation is supported by correlational links between linguistic and cognitive skills in bilinguals during language learning (e.g., Bartolotti et al., 2011; Yoshida et al., 2011) and linguistic processing (e.g., Michael and Gollan, 2005; Levy et al., 2007; Kroll et al., 2008; Linck et al., 2008, 2012; Macizo et al., 2010; Martín et al., 2010; Gollan et al., 2011a; Morales et al., 2011; Prior and Gollan, 2011; Misra et al., 2012; Pivneva et al., 2012). Bilinguals may recruit more cognitive skills than monolinguals because they typically experience interference from their dominant language during both learning and processing (e.g., Green, 1998; Linck et al., 2009), with representations from the dominant language active and competing for selection against representations in the weaker language. Therefore, bilingual learning and processing may provide training grounds for the efficient deployment of competition resolution mechanisms. To more fully understand the origin and nature of bilingual advantages, their scope must be better defined across learning and processing contexts. In the present study, we aimed to further delineate the scope of bilingual advantages across a combined nonlinguistic learning and competition resolution task. The task was analogous to auditory word recognition and required participants to map tones to visual symbols.

In constructing such a novel learning task for bilinguals and monolinguals, it was noted that these two populations may differ in how they acquire symbolic information, at least in some contexts. To create a context where bilinguals and monolinguals have equivalent experience and proficiency for processing, any differences in the acquisition of novel information must also be considered. Early sequential bilinguals have been found to outperform monolinguals at acquiring novel words, with more novel words correctly recalled after training in bilinguals than monolinguals (e.g., Van Hell and Mahn, 1997; Kaushanskaya and Marian, 2009). For example, Kaushanskaya and Marian (2009) found word learning advantages for Spanish-English and Mandarin-English bilinguals, relative to monolinguals. These enhanced bilingual word learning skills maintained at various levels of phonological familiarity (Kaushanskaya, 2012) but were limited to concrete words, suggesting that semantic content may mediate bilingual word learning (Kaushanskaya and Rechtzigel, 2012; Hemsley et al., 2013). This previous work suggests that bilinguals may be particularly successful word learners when they can rely on robust aspects of their previously-established linguistic systems (e.g., a shared semantic system, Van Hell and De Groot, 1998).

Learning outcomes may also rely on other previous linguistic skills that are not linked to bilingualism *per se*. Findings from monolingual children suggest that word learning success is positively correlated with previous word knowledge (e.g., Ellis Weismer and Evans, 2002; Gray, 2004). In bilingual children, Kan and Kohnert (2012) identified positive correlations between fast mapping success on novel words and previous vocabulary knowledge, particularly in children's weaker L2 but also in L1. Further, Kan (2014) found that bilingual children had better long-term retention of new words in their more proficient L1 than in their L2. In adult learners, positive correlations have also been found between linguistic knowledge in L1 and L2 (e.g., Marian et al., 2007; Sparks, 2009; Djigunovic, 2010), suggesting that L1 may provide facilitative scaffolding for novel L2 learning through positive transfer of knowledge (Cummins, 1979; Proctor et al., 2010).

While previous linguistic knowledge can support novel learning when skills can be transferred, bilingual experience has also been associated with better learning outcomes on artificial languages that bear no similarity to natural languages. For example, Bartolotti et al. (2011) showed better learning outcomes on a Morse code task in participants with extensive vs. minimal bilingual experience. In part, such advantages may relate to more robust auditory encoding in bilinguals than monolinguals in the presence of distractors (Krizman et al., 2012). It is thus possible that bilinguals' competition resolution skills can influence learning in a novel (nonlinguistic) domain. Bilinguals' competition resolution skills may also influence how they *process* a newly acquired code. For example, Bartolotti and Marian (2012) trained monolinguals and Spanish-English bilinguals to criterion on novel words that matched Spanish and English phonotactics. They then examined cross-linguistic competition with similar-sounding English words during comprehension in the novel language. While no bilingual learning advantage emerged, Bartolotti and Marian found that monolinguals experienced greater interference from English competitors and took longer to resolve this competition. In short, bilingual-monolingual differences have emerged both during learning and during processing of newly-learned linguistic material, and processing differences can exist even in the absence of learning differences. Therefore, examining learning success in tandem with subsequent processing may provide valuable insights into the nature of cognitive differences between bilinguals and monolinguals.

We aimed to develop learning and processing tasks that were cognitively analogous to natural language processing. Auditory word identification is one processing context where bilinguals must resolve more competition than monolinguals. During monolingual word recognition, acoustic input is mapped onto multiple word candidates and, as enough acoustic detail accrues, the target word is selected among similar-sounding alternatives. For example, while hearing *cat*, participants may also activate the word *cab* because of word-initial phonological overlap (McClelland and Elman, 1986; Marslen-Wilson, 1987). Bilinguals experience competition not only within-language (e.g., *cab-cat*) but also from between-language competitors (e.g., Marian and Spivey, 2003; Blumenfeld and Marian, 2007, 2013). For example, upon hearing the word *marbles*, a Spanish-English bilingual may also look at a picture of a butterfly (*mariposa* in Spanish). Looks to target (*marble*) vs. competitor (*mariposa*) pictures in such visual world paradigms have been linked to processing of referents in real-time (Tanenhaus et al., 2000), and although such paradigms only simulate natural multiple-competitor linguistic environments, they have been found to do so reliably (e.g., Shook and Marian, 2013), with sensitivity to the magnitude of variable competition effects across contexts (e.g., Dahan et al., 2001) and groups (e.g., Yee et al., 2008). Recent findings suggest that bilinguals' co-activation of cross-linguistic competitors (e.g., *mariposa*) during early auditory processing is modulated by cognitive control skills, where more efficient inhibitory control is associated with more efficient resolution of cross-linguistic competition (Blumenfeld and Marian, 2013; Mercier et al., 2014; Giezen et al., under review).

Given bilinguals' competition resolution demands and recruitment of cognitive control during online receptive processing, they may also apply cognitive control along a different *time course* than monolinguals (e.g., Treccani et al., 2009; Martín et al., 2010; Blumenfeld and Marian, 2011). During auditory word identification, Blumenfeld and Marian (2011) found that monolinguals continued to inhibit phonological competitors 500 ms after auditory word recognition. Instead, bilinguals with efficient nonverbal Stroop inhibition skills showed no residual inhibition of competitors 500 ms after word identification. Lifting of inhibition has been shown to take time (e.g., during bilinguals' language switches, particularly from less to more proficient languages, Meuter and Allport, 1999). Yet, bilinguals have been found to have smaller switching costs than monolinguals in the nonlinguistic domain (Prior and MacWhinney, 2009). Higher proficiency, in such cases, may increase the efficiency with which bilinguals are able to disengage attention from irrelevant cues (for an illustration of such earlier disengagement in the nonlinguistic visual domain, see Mishra et al., 2012). Thus, when bilinguals inhibit words from an irrelevant language to process the target language, the ability to rapidly apply and release inhibition may be particularly important because a non-target language may become relevant during a language switch. Nevertheless, bilinguals may also apply more prolonged inhibition than monolinguals in some nonlinguistic environments: On a nonlinguistic visual priming task that probed suppression of irrelevant information 350 ms after competing visual stimuli had been presented, inhibition effects were found for bilinguals but not monolinguals (Treccani et al., 2009). To understand bilingual-monolingual differences in the time course of inhibition, additional work is needed taking into account factors such as experience and task. Examination of inhibitory control across the time course of competition resolution provides a context to help identify how cognitive control may be modulated for bilingual processing.

While emerging findings suggest subtle bilingual-monolingual differences during nonlinguistic competition resolution, when comparing *within-language* competition resolution in bilinguals vs. monolinguals (e.g., Marian and Spivey, 2003; Blumenfeld and Marian, 2011), effects may be partially driven by inherent group differences in language experience and proficiency. Even in their native language, bilinguals and monolinguals have been shown to differ in word retrieval (e.g., Ivanova and Costa, 2008). Such bilingual retrieval disadvantages have been ascribed to weaker links between form and meaning representations due to less time spent in each of bilinguals' languages (Gollan et al., 2011b) and have been shown in both comprehension and production (Duyck et al., 2008; Gollan et al., 2011b). As such, underlying task demands, and strategies to meet them, may differ across groups, and may in turn drive differences in competition resolution. One alternative to examining language-based processing is to create nonlinguistic analog tasks that match language-based competition resolution in terms of cognitive processes involved, while ensuring equal exposure to the task across the two groups.

In the current study, we compared bilinguals to monolinguals on a nonlinguistic learning task. Participants acquired a novel symbolic system, requiring information processing that is analogous to cognitive processes of auditory word identification. The phonological characteristics of the novel symbolic system had no overlap with already-known languages, allowing equal footing between groups in terms of content knowledge. Participants were taught twelve tone-to-symbol mappings, consisting of combinations of three feature contrasts (timbre, pitch, duration). Since differences in cross-linguistic competition may be one reason for different learning and processing outcomes in bilinguals and monolinguals, the current symbolic system was devised to *not* elicit cross-linguistic interference or facilitation with already-known languages. However, a critical aspect of natural spoken languages was maintained: sound-based similarity between items, which can create temporal ambiguities that must be resolved prior to word/symbol identification. Further, the tone-to-symbol mapping task was structurally similar to linguistic word learning, which has been argued to involve familiarization with a new word form (a tone in this case), a novel referent (a symbol in this case), as well as creating an associative link between the word form and the referent (e.g., Storkel, 2001; Kan, 2014).

After learning was completed, processing of tone-to-symbol mappings was examined by presenting participants with displays of four symbols together with one auditory tone. Participants were instructed to identify the symbol corresponding to the tone from among four symbols, with temporal ambiguity present between the target item and a competitor item. This processing task is analogous to previous research on auditory word identification (e.g., Marian and Spivey, 2003; Blumenfeld and Marian, 2007, 2011, 2013; Mercier et al., 2014). Just as the difference between *cat* and *cap* is resolved based on auditory information in the final phoneme, so the target and competitor tones were identical on all dimensions except the duration of the tone. In both cases, participants were given similar “top down” information from visual displays, with two similar-sounding items on critical trials that could only be distinguished once an auditory uniqueness point was reached. In addition, a previously-developed paradigm was employed where symbol identification trials were followed by location priming probes that indexed residual activation of target locations and residual inhibition of competitor locations (Blumenfeld and Marian, 2011). Based on previous research on time course differences in bilingual and monolingual inhibition (Treccani et al., 2009; Blumenfeld and Marian, 2011), residual facilitation and inhibition were examined 200, 500, and 800 ms postsymbol-identification.

On the *learning component* of the task, we predicted no or limited bilingual advantages since the task did not rely on previously learned conceptual structures (Kaushanskaya and Rechtzigel, 2012). If bilingual advantages were to arise, we predicted that they would originate from differences in auditory encoding (Bartolotti et al., 2011; Krizman et al., 2012) and/or from mastering the novel combinatorial system of auditory-visual features. Second, we predicted that bilinguals and monolinguals who had mastered the learning task equally well would show subtle *processing differences* as indexed by target identification performance and by the time course of continued target activation and competitor inhibition, with potentially smaller competition effects and different timing of inhibition release in bilinguals, as revealed in previous nonlinguistic studies (e.g., Bialystok et al., 2004; Costa et al., 2008; Treccani et al., 2009; Mishra et al., 2012). We thus expected the learning and processing aspects to be potentially independent as far as effects of bilingualism were concerned. Identification of bilingual-monolingual processing differences in groups matched on experience would strengthen the case for underlying bilingual-monolingual differences in the engagement of cognitive processes. Finally, we predicted that including success during the symbolic learning task and previous vocabulary knowledge as explanatory variables would reveal their influence during processing, with both measures potentially related to more efficient performance.

## Methods

### Participants

Thirty-eight Spanish-English bilinguals (2 males, mean age = 21.3, *SD* = 2.9) and 29 English monolinguals (10 males, mean age = 23.0, *SD* = 2.9) were originally recruited to participate. The two groups were similar in years of formal education (monolinguals: 15.2 years, *SD* = 2.3; bilinguals: 15.5 years, *SD* = 1.9), *t*_(65)_ = −0.58, *p* > 0.5, verbal working memory as measured by a numbers reversed task (monolinguals: 17.4, *SD* = 4.5; bilinguals: 16.2, *SD* = 4.0), *t*_(65)_ = 1.2, *p* > 0.1, and nonverbal reasoning as indexed by the matrix reasoning subtest of the Wechsler Abbreviated Scale of Intelligence (WASI) (monolinguals: *T*-score = 56.6, *SD* = 12.5; bilinguals: *T*-score = 52.4, *SD* = 6.6), *t*_(65)_ = 1.6, *p* > 0.1. However, bilinguals and monolinguals differed in terms of English receptive vocabulary as indexed by the PPVT (monolinguals: 182.1, *SD* = 6.9; bilinguals: 176.0, *SD* = 9.5), *t*_(65)_ = 2.94, *p* = 0.005, on the Test of Auditory Discrimination (monolinguals: 56.3, *SD* = 18.3; bilinguals: 48.1, *SD* = 7.3), *t*_(65)_ = 2.5, *p* < 0.05, and on self-reported measures of language background, including proficiency in English on a scale from 0 (*none*) to 10 (*excellent*) (monolinguals: 9.6, *SD* = 0.7; bilinguals: 9.2, *SD* = 0.8), *t*_(65)_ = 2.3, *p* < 0.05. Groups also differed in their current exposure to English (monolinguals: 99.0%, *SD* = 2.2; bilinguals: 61.9%, *SD* = 20.6), *t*_(65)_ = 9.6, *p* < 0.001. Bilinguals included 19 early Spanish-English bilinguals, 6 simultaneous Spanish-English bilinguals, and 1 early English-Spanish bilingual. Bilinguals acquired Spanish at an average age of 0.2 years (*SD* = 1.0) and English at an average age of 3.7 years (*SD* = 2.8) and rated their proficiency in Spanish at 8.4 (*SD* = 1.4).

Since processing (based on accurate performance) of newly-learned symbolic information was of interest, inclusionary criteria were set so that participants were only included in analyses if their overall accuracy on symbolic learning and processing tasks was at least 65%, a threshold that was chosen based on the distribution of learning and processing accuracies across all participants. Two monolinguals and 11 bilinguals were omitted from primary analyses because their overall (post-training and processing) accuracies on tone-to-symbol matching were below 65% (excluded monolinguals: mean score = 49.0%, range = 34.0–64.1%; excluded bilinguals: mean score = 49.5%, range = 28.1–58.6%). This resulted in a total of 27 monolinguals and 27 bilinguals included in analyses on learning and processing of novel symbolic information (for details, see Table 1). All participants passed a hearing screening at 20 dB at 500, 1000, 2000, and 4000 Hz (American Speech-Language-Hearing Association, 1997). The study was conducted with approval of the San Diego State University Institutional Review Board and following all regulatory standards.

**Table 1:** **Bilingual and monolingual participants included in primary analyses of sound-to-symbol processing**.

	**Bilinguals *n* = 27 *M* (*SD*)**	**Monolinguals *n* = 27 *M* (*SD*)**	***t*_(52)_, *p***
Age	21.3 (3.1)	22.8 (2.9)	1.9, >0.05
Years of formal education	15.5 (2.0)	15.3 (2.3)	−0.3, >0.5
PPVT, English receptive vocab (raw score)	177.0 (10.5)	182.2 (7.0)	2.1, 0.04
WASI matrix reasoning *T*-score	53.1 (6.2)	55.9 (12.7)	1.0, >0.1
Test of auditory discrimination	47.3 (7.5)	55.2 (17.5)	2.2, 0.04
Numbers reversed	16.9 (3.8)	17.5 (4.7)	0.5, >0.5
TVIP, Spanish receptive vocab (raw score)	105.7 (8.4)	N/A	N/A
Age of English acquisition	3.7 (2.9)	0.0 (0.0)	−5.9, <0.001
Age of English fluency	6.6 (3.4)	3.5 (2.2)	−4.0, <0.001
Percentage current exposure to English	61.7 (19.4)	98.9 (2.3)	9.9, <0.001
Self-reported proficiency speaking English	9.3 (0.7)	9.6 (0.7)	1.7, 0.09
Age of Spanish acquisition^a^	0.2 (1.2)	15.4 (3.9)	18.7, <0.001
Age of Spanish fluency	4.6 (4.2)	N/A	N/A
Percentage exposure to Spanish^a^	37.0 (18.6)	0.7 (1.4)	−10.1, <0.001
Self-reported proficiency speaking Spanish^a^	8.3 (1.5)	1.6 (1.5)	−16.2, <0.001

PPVT, Peabody Picture Vocabulary Task; WASI, Wechsler Abbreviated Scale of Intelligence.

a On these rows, values entered for monolinguals correspond to a later-acquired L2 (Spanish or other).

### Design

The learning component of the experiment included group (bilingual, monolingual) as a between-subjects variable. The picture identification component of the experiment followed a 2 × 2 design, with picture condition (competitor trial, non-competitor trial) as a within-subject variable, and with group (bilingual, monolingual) as a between-subjects variable. The priming component followed a 3 × 3 × 2 design, with priming condition (target probe, competitor probe, baseline probe) and stimulus-onset asynchrony between picture and priming trials (200, 500, 800 ms) as within-subject variables, and with group (bilingual, monolingual) as a between-subjects variable.

### Materials

#### Experimental materials

**Figure 1:**
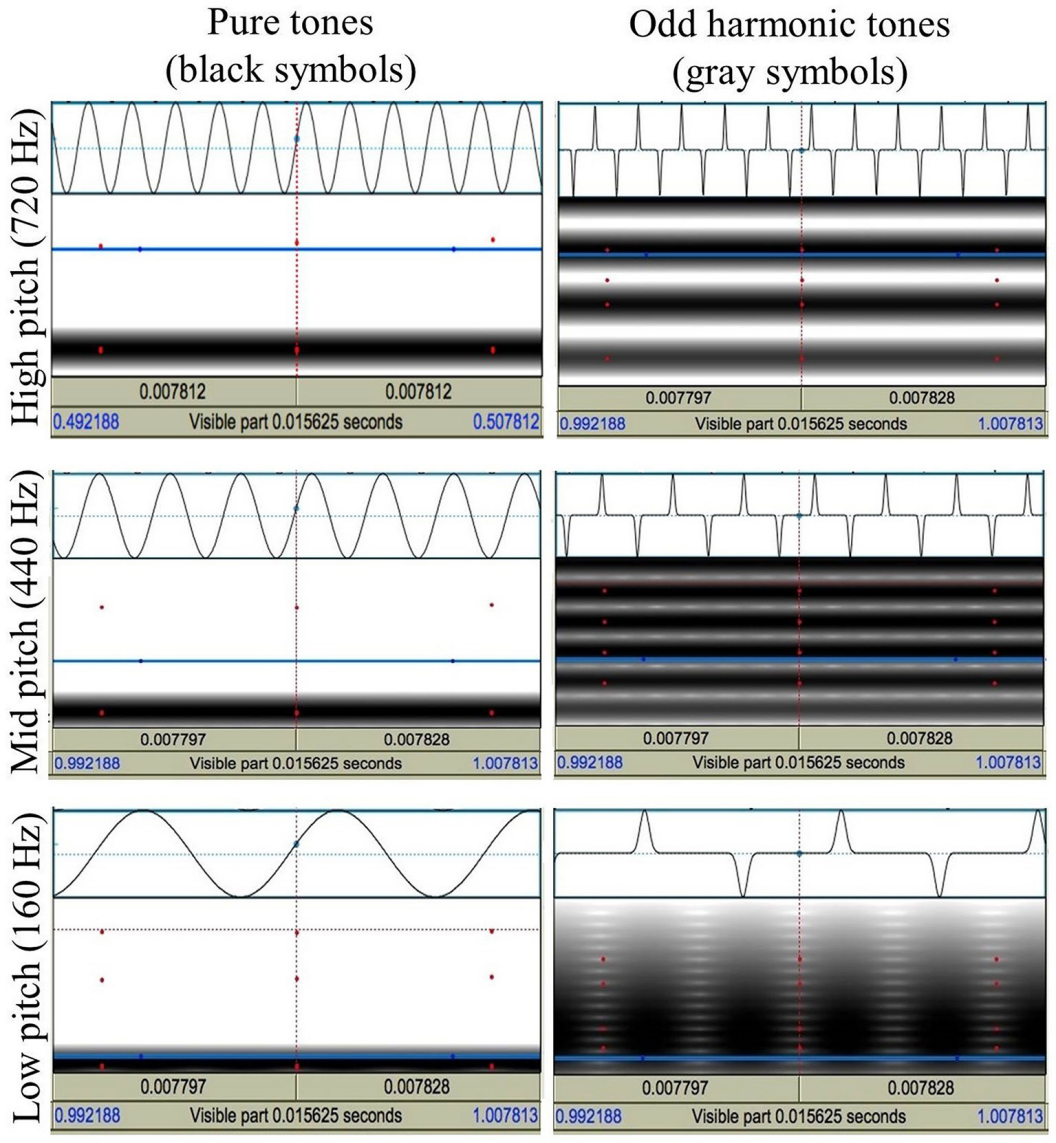
**Waveform and spectrogram excerpts describing the pitch (720, 440, 160 Hz) and timbre (pure tone, odd harmonic tone) characteristics of the sound stimuli**. Images generated in Praat (Boersma and Weenink, 2014).

Twelve tones were created that differed on three dimensions: timbre (pure tones vs. tones containing odd harmonics yielding a more hollow sound), pitch (160, 440, 720 Hz), and tone length (400, 1000 ms). A pure sine wave tone (without harmonics) and a squared sine wave tone (with odd harmonics) were chosen to be types of tones that were easily distinguishable in timbre, given initial feedback from six untrained listeners from the same population as the research participants. All tones were generated using Adobe Audition software. For waveform and spectrogram excerpts describing the tones' pitch and timbre characteristics, see Figure 1.

Artificial languages that rely on perception of duration have previously been learned successfully. For example, Bartolotti et al. (2011) taught bilingual and monolingual participants an artificial language that required mastering a contrast between tones of 100 ms vs. 300 ms in duration. Similarly, previous research suggests that training can improve discrimination accuracy of a 200 Hz frequency distinction at 200, 360, and 2500 Hz (Demany, 1985; for similar findings, see Carcagno and Plack, 2011), suggesting trainability within our chosen range of frequencies. Finally, the minimum length of a tone necessary to perceive a distinct timbre varies between 10 and 200 ms depending on stimulus frequency (Wang, 1983), and the length of our tones was chosen to allow for robust timbre distinctions. While previous findings and pilot data suggested that each contrast (pitch, timbre, duration) could be mastered, it was expected that the combination of these dimensions would render learning and processing of stimuli more challenging (e.g., Krumanshl and Iverson, 1992). The difficulty of the novel learning task would therefore ensure that participants with various success levels would emerge.

**Figure 2:**
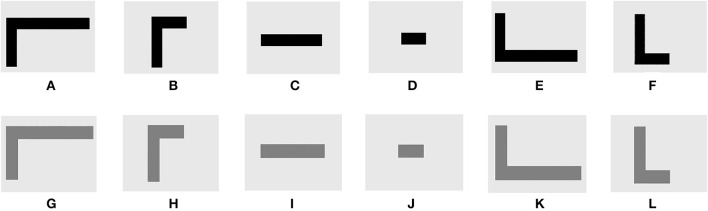
**Symbols that correspond to the 12 trained tones**. Symbol-to-tone correspondences include **(A)** *High long black*: high-pitched 1000 ms sine wave; **(B)** *High short black*: high-pitched 400 ms sine wave; **(C)** *Mid long black*: mid-pitched 1000 ms sine wave; **(D)** *Mid short black*: mid-pitched 400 ms sine wave; **(E)** *Low long black*: low-pitched 1000 ms sine wave; **(F)** *Low short black*: low-pitched 400 ms sine wave; **(G)** *High long gray*: high-pitched 1000 ms harmonic wave; **(H)** *High short gray*: high-pitched 400 ms harmonic wave; **(I)** *Mid long gray*: mid-pitched 1000 ms harmonic wave; **(J)** *Mid short gray*: mid-pitched 400 ms harmonic wave; **(K)** *Low long gray*: low-pitched 1000 ms harmonic wave; **(L)** *Low short gray*: low-pitched 400 ms harmonic wave.

Twelve picture symbols to correspond to the 12 tones were created using Paint and Adobe Photoshop software (see Figure 2). The pictures varied along the same three dimensions as the tones, with black shapes representing the sine wave tones and gray shapes representing the squared sine wave tones (see Figures 2A–F vs. G–L). Mid-pitch tones were represented by stand-alone horizontal lines (Figures 2C,D,I,J); high-pitch tones were represented by raised horizontal lines attached to the top of vertical lines (Figures 2A,B,G,H); low-pitch tones were represented by a lowered horizontal line attached to the bottom of a vertical line (Figures 2E,F,K,L). Finally, longer tones were represented by longer lines (Figures 2A,C,E,G,I,K) and shorter tones were represented by shorter lines (Figures 2B,D,F,H,J,L).

**Figure 3:**
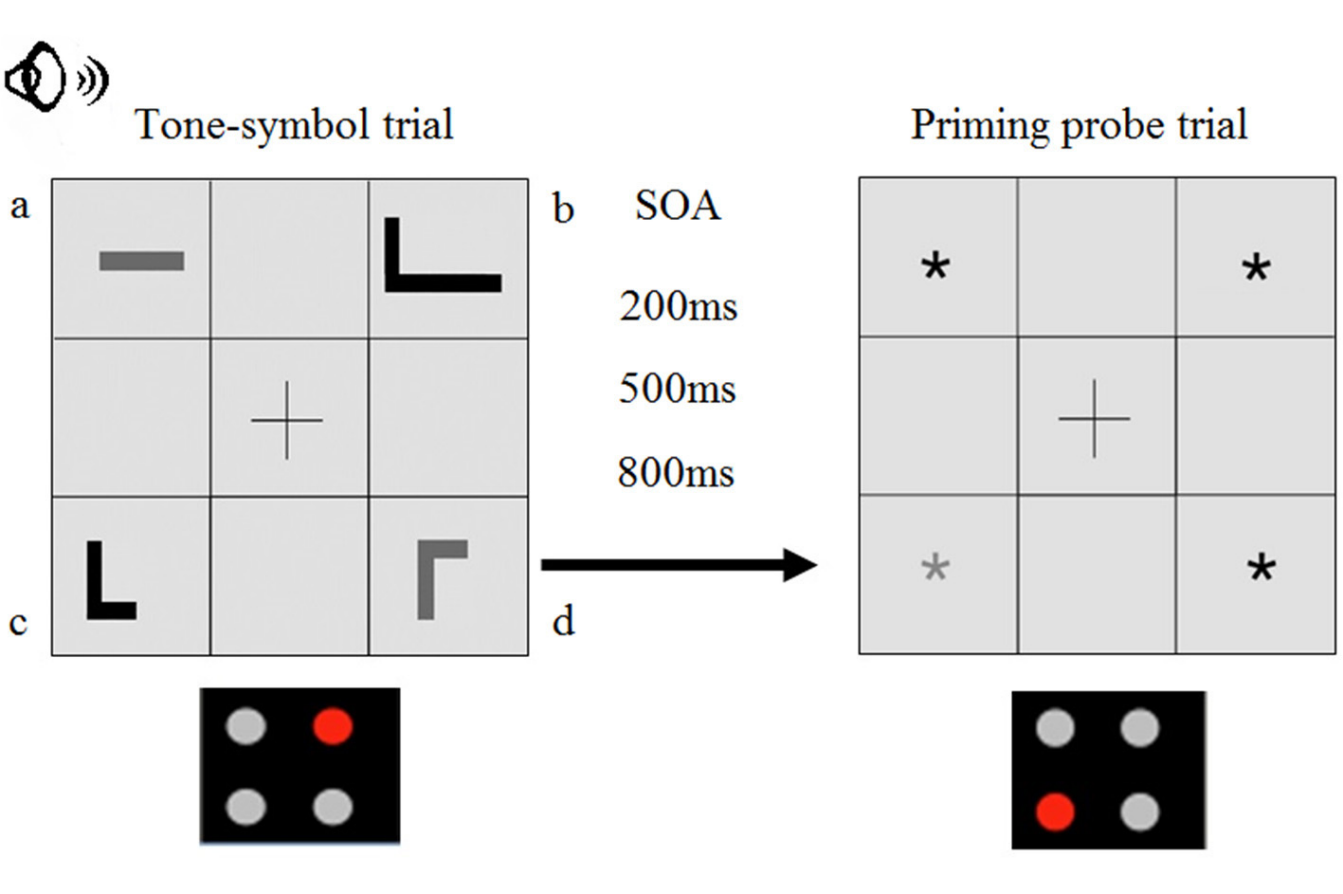
**Sample trial display, including a tone-symbol trial followed by a priming probe trial**. For example, in this trial, the target could be the *low long black* symbol (b), with the participant hearing the *low long black* (160 Hz 1000 ms sine wave) tone. For the first 400 ms, this tone would also match the *low short black* symbol (i.e., the competitor, c). Shapes in the top left and bottom right quadrants contain symbols that are easily ruled out based on pitch and timbre (a,d). Since the gray asterisk appears in the location previously occupied by the competitor (c), the priming probe is a negative priming trial. Participants identified the gray asterisk from among the black asterisks.

Visual stimulus displays were created that contained four of the picture symbols, one in each quadrant on the display (see Figure 3). Each critical display included a target symbol, a competitor symbol that was identical to the target in terms of timbre and pitch but differed in duration, and two filler symbols that differed from target and competitor shapes in terms of timbre and pitch. As a result, target and competitor pictures were easily distinguishable from filler pictures relatively early after the onset of the tone, with previous research suggesting that pitch and timbre contrasts can be made within 200 ms (e.g., Wang, 1983). Conversely, target and competitor pictures were distinguishable only after 400 ms, since they differed only in length. Given that separation from filler symbols in this combinatorial system would take about 200 ms, participants were expected to identify targets and competitors (vs. fillers) after the first 200 ms, followed by an additional 200 ms of ambiguity between targets and competitors. A 200 ms ambiguity is comparable to the phonological overlap between words (e.g., *cat*-*cab*) in previous linguistic studies (e.g., Blumenfeld and Marian, 2013: *M* = 251 ms of cross-linguistic ambiguity, *SE* = 31; Blumenfeld and Marian, 2011: *M* = 279 ms of within-language ambiguity, *SE* = 21). Across the critical trials, each stimulus type (target, competitor, filler) occurred in each quadrant an equal number of times.

Participant responses to the tone-symbol stimulus were followed by silence that lasted 200, 500, or 800 ms, after which participants were presented with a priming probe display that contained four asterisks, one in each quadrant. Three of the asterisks were black and one was gray. Participants' response speed to the gray asterisk indexed residual facilitation or inhibition of the preceding tone-symbol stimulus. Specifically, if the gray asterisk appeared in the same quadrant position as the previous tone-symbol target, then it would index residual facilitation given previous activation of the target (i.e., it would be a positive priming trial). If the gray asterisk appeared in the same position as the previous tone-symbol competitor, then it would index residual inhibition of the competitor (i.e., it would be a negative priming trial). Finally, if the gray asterisk appeared in the same quadrant as one of the two previous filler pictures, it would index neither facilitation nor inhibition (i.e., it would be a baseline trial). To summarize, if the gray asterisk was placed in the quadrant that had previously been occupied by the target picture, then a quicker identification time was expected if the quadrant had previously received attention. If the gray asterisk was placed in the quadrant that had previously been occupied by the competitor picture, then a delay was expected if this picture had previously been inhibited. Finally, both competitor inhibition and target facilitation were measured relative to a neutral baseline, where gray asterisks were placed in quadrants that had previously been occupied by neutral filler items. Different inter-stimulus intervals (200, 500, or 800 ms) between the tone-symbol trials and the gray asterisk trials probed how long facilitation and inhibition stayed active. The experiment script was prepared using SuperLab experimental software (Cedrus, Phoenix, Arizona). The gray asterisk occurred in each quadrant an equal number of times for each priming condition (target, competitor, baseline).

#### Training of tone-to-symbol mappings

**Figure 4:**
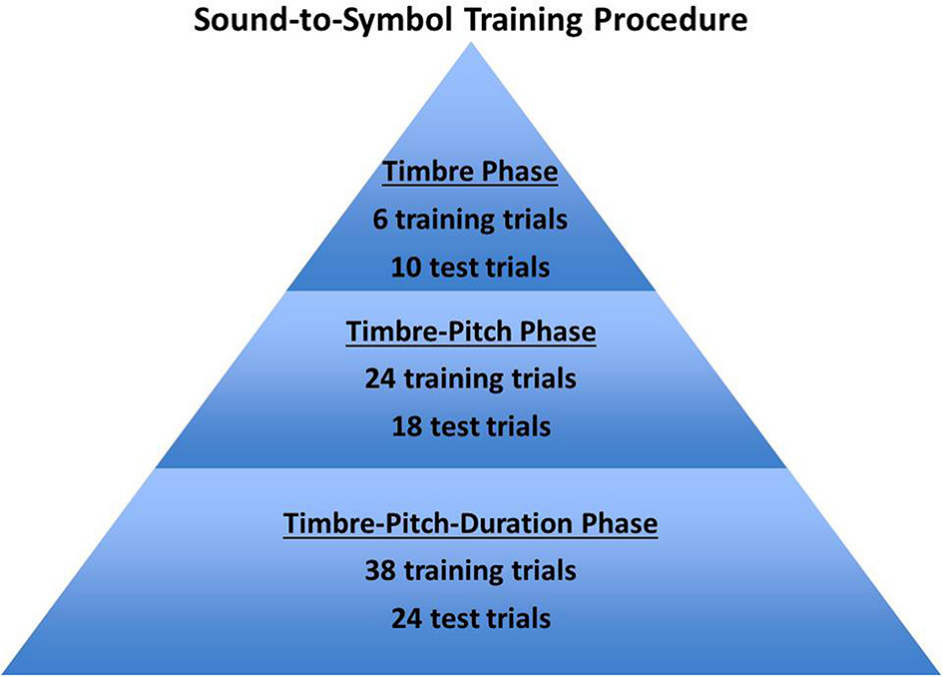
**Summary of the sound-to-symbol training procedure to familiarize participants with the sound-to-symbol mappings, involving timbre, pitch, and duration dimensions**.

Participants underwent one training session of approximately 30 min, administered immediately before they participated in the processing experiment (for an illustration of the three training phases, see Figure 4). They were first trained on *timbre* contrasts only, using gray and black long symbol images. A total of six timbre training trials were presented using the 440 Hz/1000 ms tones of both the pure sine wave (three trials) and odd-harmonic wave (three trials), so that participants learned to associate the two timbres with their corresponding black and gray symbols. During this training phase, participants were presented with two symbols, one black and one gray, and asked to choose the symbol that corresponded to the tone they heard. Following the timbre training, participants were presented with 10 timbre test trials, where they heard a tone and had a choice between two symbols. If participants answered correctly they saw a screen with the chosen symbol reading “that's correct”; if they answered incorrectly, they saw a screen reading “that's incorrect. The correct answer is,” followed by the correct target symbol. During the next training segment, participants were trained on *pitch*, using different timbre combinations. These were presented in sets of three: first the low-, mid-, and high-pitched tones of the timbre associated with black symbols, followed by the low-, mid-, and high-pitched tones of the timbre associated with gray symbols. This cycle repeated four times for a total of 24 pitch-timbre training trials. In the pitch-timbre test phase, participants were asked to identify the symbol that matched the tone they heard out of a field of four symbols, all of 1000 ms duration, and were provided feedback. Eighteen pitch-timbre test trials were presented, such that participants heard each pitch and timbre combination three times. Finally, participants were trained on the *duration* contrast, using different pitch and timbre combinations. Thirty-eight training trials were presented combining duration, pitch, and timbre. The first six were of the pure tone timbre (that is, a short and long tone of each of the three pitches) and the second six were of the odd-harmonic tone timbre. The last 26 training trials were presented in random order to approximate the presentation that would take place in the actual task. Participants were then presented with a final 24-trial test phase in which they again saw displays as in Figure 2 and were asked to press the button on the keyboard that corresponded to the symbol matching the tone they heard. To familiarize participants with the task set-up, each symbol matching trial during the final test phase was followed by an asterisk identification trial, for which participants were asked to indicate the location of the gray asterisk (through the use of the same keyboard buttons as in the symbol match task, see Figure 3). For examples of training trials, see Appendix A.

#### Processing of tone-to-symbol mappings

The processing experiment included three blocks, one for each inter-stimulus interval (200, 500, 800). Block order was counterbalanced across participants. Each block consisted of 144 trials, 72 of which were critical trials. In all 72 critical tone-to-symbol trials, a target was presented together with a competitor item. On 24 of these trials, subsequent gray asterisks appeared in the location on the display that had previously been occupied by the target (positive priming trials); on 24 trials, subsequent gray asterisks appeared in the location previously occupied by the competitor (negative priming trials); finally, on 24 trials, subsequent gray asterisks appeared in the location previously occupied by one of the filler items (baseline priming trials). The remaining 72 trials were designed to be baseline trials where no competitor was present. The purpose of these trials was 2-fold: (1) to provide a no-competition baseline for analyses of competition effects during tone-to-image mapping; and (2) to avoid participant bias during target selection toward one of the overlapping items on the display. In order to accomplish this, no competitor was present but two symbols were present that overlapped with one another in terms of timbre and pitch but not duration, with neither of these items being the target during tone-to-symbol matching. Thus, when these trials were presented together with an auditory target, the auditory target was more readily identified without extended competition from another image.

#### Linguistic and cognitive assessments

The *Language Experience and Proficiency Questionnaire* was administered to capture demographic information as well as language learning history and self-reported proficiency (Marian et al., 2007). Cognitive skills were assessed with the matrix reasoning sub-test of the WASI, which has been found to correlate highly with overall nonverbal IQ (WASI, Wechsler, 1999). In addition, verbal working memory was indexed using the numbers reversed sub-test of the Woodcock-Johnson *Tests of Cognitive Abilities III* (Schrank et al., 2001), and English auditory phoneme discrimination skills were examined using the quiet section of the *Test of Auditory Discrimination*, where participants had to distinguish between minimal pairs (e.g., *fair*-*pear*, Goldman et al., 1970). In addition, participants completed a nonlinguistic Stroop task to index nonverbal inhibition skills. On this task, participants were instructed to indicate the direction of an arrow as pointing right or left by hitting response keys with their right or left index fingers, respectively. Arrows were presented either centrally on a screen, to the right, or to the left. In instances where right-pointing arrows were presented on the left or left-pointing arrows were presented on the right, participants had to inhibit location information in order to make efficient correct responses. This task matches the classic Stroop task in terms of task components and type of inhibition required (Kornblum, 1994; for more detailed description of task characteristics, see Blumenfeld and Marian, 2014, Experiment 2). Finally, receptive vocabulary skills were assessed with the *Peabody Picture Vocabulary Task, third edition* in English (PPVT, Dunn and Dunn, 1997) and the *Test de Vocabulario en Imagenes Peabody* in Spanish (TVIP, Dunn et al., 1986).

### Procedure

Participants first completed informed consent, the *Language Experience and Proficiency Questionnaire*, and a hearing screening, followed by cognitive tests and language tests within each language separately. Participants were then seated in front of a computer in a quiet environment and were instructed to follow training procedures for the tone-to-symbol learning stage of the study. Auditory stimuli were presented at a volume that was comfortable for the participant.

After participants completed the training session, they started the experimental session where processing of newly-learned tone-symbol correspondences was examined. Participants were asked to look at displays containing four quadrants and, 500 ms after appearance of each display, an auditory tone was presented. Participants were instructed to promptly identify which picture corresponded to the tone that was presented by using labeled keys on the keyboard that corresponded to the location of the quadrant (i.e., top left: key “e”; top right: key “o,” pressed with the index fingers of the left and right hand, respectively; bottom left: key “c”; bottom right: key “m,” pressed with the thumbs of the left and right hand, respectively). The keys were clearly marked with different colors, and participants maintained their fingers positioned on the four keys throughout the task to ensure rapid responses. In the following trials, participants were instructed to identify the gray asterisk as promptly as possible by using the same keys on the keyboard.

### Data coding and analyses

Accuracies and reaction times were obtained via SuperLab software. For the *training component* of the study, accuracies and reaction times were compared across groups. To examine overall performance without the influence of speed-accuracy trade-offs, combined scores of accuracy and reaction times were also derived (efficiency scores, i.e., reaction times divided by proportion correct, e.g., Townsend and Ashby, 1983). For all analyses, responses were included for correct trials only. Responses for priming probe trials were only included if responses were correct for the preceding tone-to-symbol trial *and* the priming probe trial. For the processing component of the study, analyses are presented only for efficiency scores for simplicity, given similar performance patterns across accuracy and reaction time variables. Mixed linear models were employed to examine target identification and competition resolution in bilinguals and monolinguals, using the MIXED procedure in SPSS. Within this procedure, maximal random effects structures were considered (Barr et al., 2013), and structures were simplified until each model converged. Categorical variables were deviation coded in all analyses. For target identification, “target type” was entered into the random effects structure, given that participants were asked to respond to one of twelve sound-to-symbol matches across trials. For priming probe analyses, “target type” was not considered because participants consistently responded to gray asterisks. Since the role of previous language knowledge and learning outcomes on the training task were of particular interest, they were entered into analyses as continuous variables. These variables were centered and standardized through derivation of z-scores to reduce collinearity. Response times that were more than three standard deviations away from the mean were removed from further analyses.

## Results

### Sound-to-symbol mapping learning outcomes

When learning outcomes were examined in all bilinguals and monolinguals, including individuals who performed below 65% accuracy, statistically equivalent performance was found between bilinguals' post-training accuracy (*M* = 71.5%, *SD* = 16.3) and monolinguals' post-training accuracy (*M* = 76.1%, *SD* = 13.1), *t*_(65)_ = 1.2, *p* > 0.1. However, a chi-squared test examining the distribution of low-accuracy performers (<65%) across bilinguals and monolinguals was marginally significant, Chi-Squared (*df* = 1) = 3.8, *p* = 0.051, suggesting different distributions of lower learning outcomes in the bilinguals vs. monolinguals (see *Participants* Section). Nonparametric independent samples Mann–Whitney U tests suggested that the bilingual learners and non-learners differed on post-training and overall processing accuracies (*p*s < 0.001), showed a marginal difference on numbers reversed performance (*p* = 0.076, learners: *M* = 16.9, *SE* = 0.7; non-learners: *M* = 14.5, *SE* = 1.2), but did not differ on the remaining language history, cognitive, and linguistic measures listed in Table 1.

When participants with overall accuracy below 65% were excluded, bilinguals (*n* = 27) and monolinguals (*n* = 27) reached similar post-training accuracies (monolinguals: *M* = 77.6%, *SE* = 2.2; bilinguals: *M* = 77.1%, *SE* = 2.1), *t*_(52)_ = 0.16, *p* > 0.5, and response times (monolinguals: *M* = 3900.7 ms, *SE* = 215.4; bilinguals: *M* = 4417.8 ms, *SE* = 215.4), *t*_(52)_ = 1.7, *p* = 0.1. Examination of post-training accuracies showed that monolinguals and bilinguals had equivalent mastery of the timbre dimension [main effect of group: *F*_(1, 52)_ = 1.8, *p* > 0.1; group × timbre interaction: *F*_(1, 52)_ = 0.089, *p* > 0.5], the pitch dimension [main effect of group: *F*_(1, 52)_ = 1.8, *p* > 0.1; group × pitch interaction: *F*_(1, 52)_ = 0.05, *p* > 0.5], and the duration dimension [main effect of group: *F*_(1, 52)_ = 1.9, *p* > 0.1; group × duration interaction: *F*_(1, 52)_ = 1.4, *p* > 0.1]. These findings suggested equivalent mastery of the learned symbolic system. Further, more efficient performance on tone-to-symbol mappings (response times divided by proportion correct) was associated with higher scores on the receptive vocabulary tasks in both monolinguals (PPVT: *r* = −0.46, *p* < 0.05) and bilinguals (PPVT: *r* = −0.36, *p* = 0.063; TVIP: *r* = −0.22, *p* > 0.1; combined PPVT/TVIP: *r* = −0.34, *p* = 0.087), suggesting that previous vocabulary knowledge is positively associated with learning success in the novel symbol system in terms of both higher accuracy rates and faster retrieval times. No associations were found between learning outcomes and performance on the matrix reasoning subtest of the WASI (*p*s > 0.5), numbers reversed (*ps > 0.1)*, or on the Test of Auditory Discrimination (*ps* > 0.5).

### Processing and competition resolution during sound-to symbol mapping

#### Sound-to-symbol mapping, competition resolution and previous vocabulary knowledge

**Figure 5:**
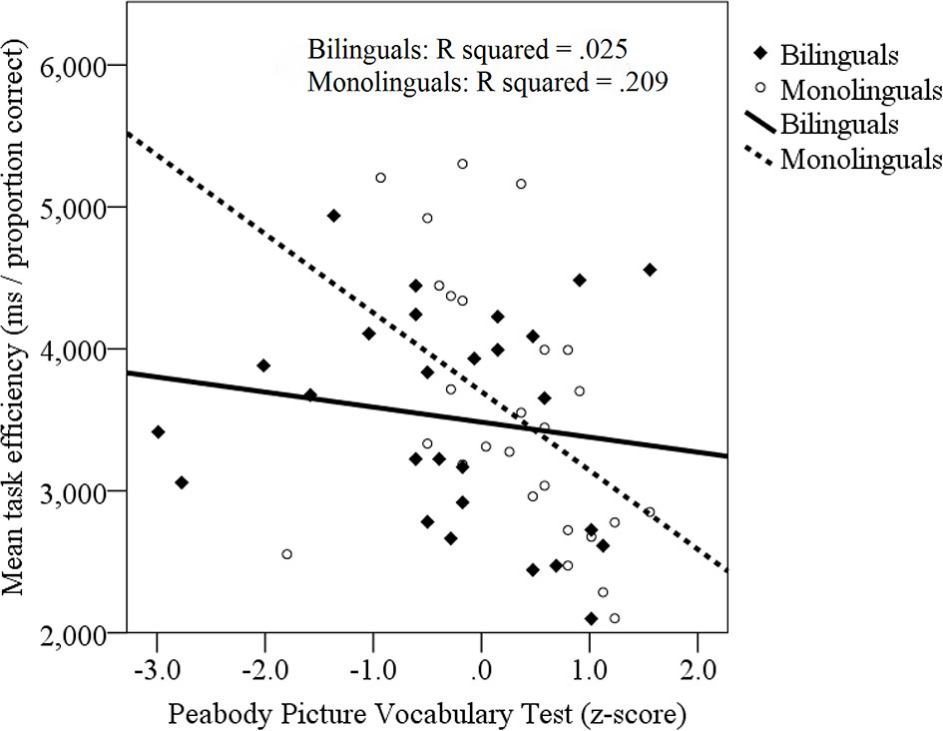
**Relationship between target identification efficiency (ms/proportion correct) during sound-to-symbol matching and participants' performance on the Peabody Picture Vocabulary Task (PPVT, z-transformed)**. Bilinguals: filled diamonds, solid line; Monolinguals: open circles, dotted line.

To examine target identification during processing of similar tone-to-symbol mappings, a mixed linear model was employed with fixed effects including trials with and without competitor symbols (competitor, filler; baseline: filler) and language group (bilingual, monolingual; baseline: monolingual). In addition, z-transformed PPVT scores were entered as a continuous predictor variable. Finally, participants and items (target type) were entered as random effects on the slope. Findings yielded a main effect of competitor, with longer and less accurate responses to competitor trials (*M* = 3663.0 ms/proportion correct, *SE* = 118.6) than to filler trials (*M* = 3388.8 ms/proportion correct, *SE* = 105.1), *b* = −608.7, *SE* = 295.5, *p* < 0.05. In addition, a main effect of vocabulary skill was found, with higher PPVT skills associated with quicker and more accurate responses (*b* = −44.6, *SE* = 295.8, *p* < 0.05). Finally, an interaction emerged between language group and PPVT, with a stronger association between target identification efficiency and PPVT performance in monolinguals (*R*^2^ = 0.209) relative to bilinguals (*R*^2^ = 0.025, see Figure 5), *b* = −980.8, *SE* = 533.8, *p* = 0.05. No other effects were significant.

To examine the possibility that receptive vocabulary in Spanish, or in Spanish and English combined, would be a better predictor of performance in bilinguals, mixed linear models were created with performance on the Spanish TVIP and with combined performance on the PPVT and TVIP. No significant effects emerged for bilinguals involving either the TVIP or the combined PPVT and TVIP (all *p*s > 0.1). Together, findings suggest that (1) competition resolution during processing of novel symbolic information was comparable across bilinguals and monolinguals, (2) previous vocabulary knowledge did not influence competition resolution, and (3) previous vocabulary knowledge *did* influence overall response efficiency on the novel processing task, but more so in monolinguals than bilinguals.

#### Sound-to-symbol mapping, competition resolution and previous success in learning symbolic information

**Figure 6:**
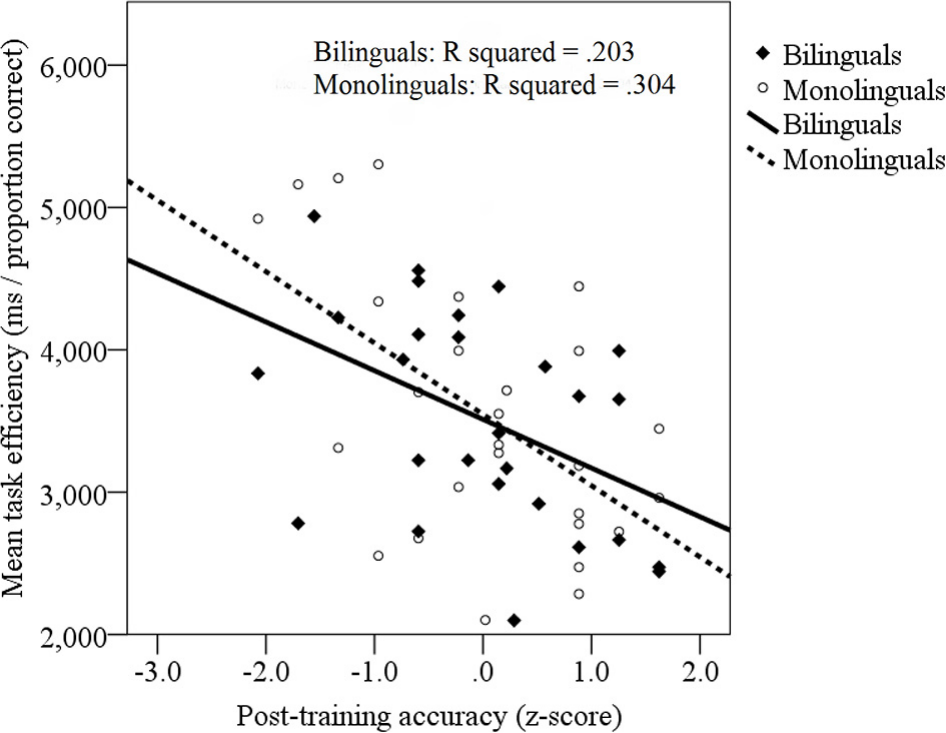
**Relationship between target identification efficiency (ms/proportion correct) during sound-to-symbol matching and participants' previous sound-to-symbol learning success (accuracy, z-transformed)**. Bilinguals: filled diamonds, solid line; Monolinguals: open circles, dotted line.

To examine the influence of learning success on target identification efficiency and competition resolution, previous symbolic learning success (z-transformed post-training accuracy) was entered into the previously-described mixed linear model instead of vocabulary skill. In addition to the previously-described main effect of competitor (*b* = −600.9, *SE* = 286.6, *p* < 0.05), a main effect of training success was identified: Greater learning success was associated with greater tone-to-symbol retrieval efficiency, *b* = −820.3, *SE* = 307.4, *p* < 0.01. This pattern was of statistically equivalent magnitude in bilinguals (*R*^2^ = 0.203) and monolinguals (*R*^2^ = 0.304), see Figure 6. No other effects were significant. Thus, (1) previous learning success did not influence competition resolution, yet (2) previous learning success *did* influence overall response efficiency on the novel processing task, an effect that did not differ across monolinguals and bilinguals.

### Priming: residual activation of sound-to-symbol targets and competitors

#### Residual inhibition of competitor symbols

**Figure 7:**
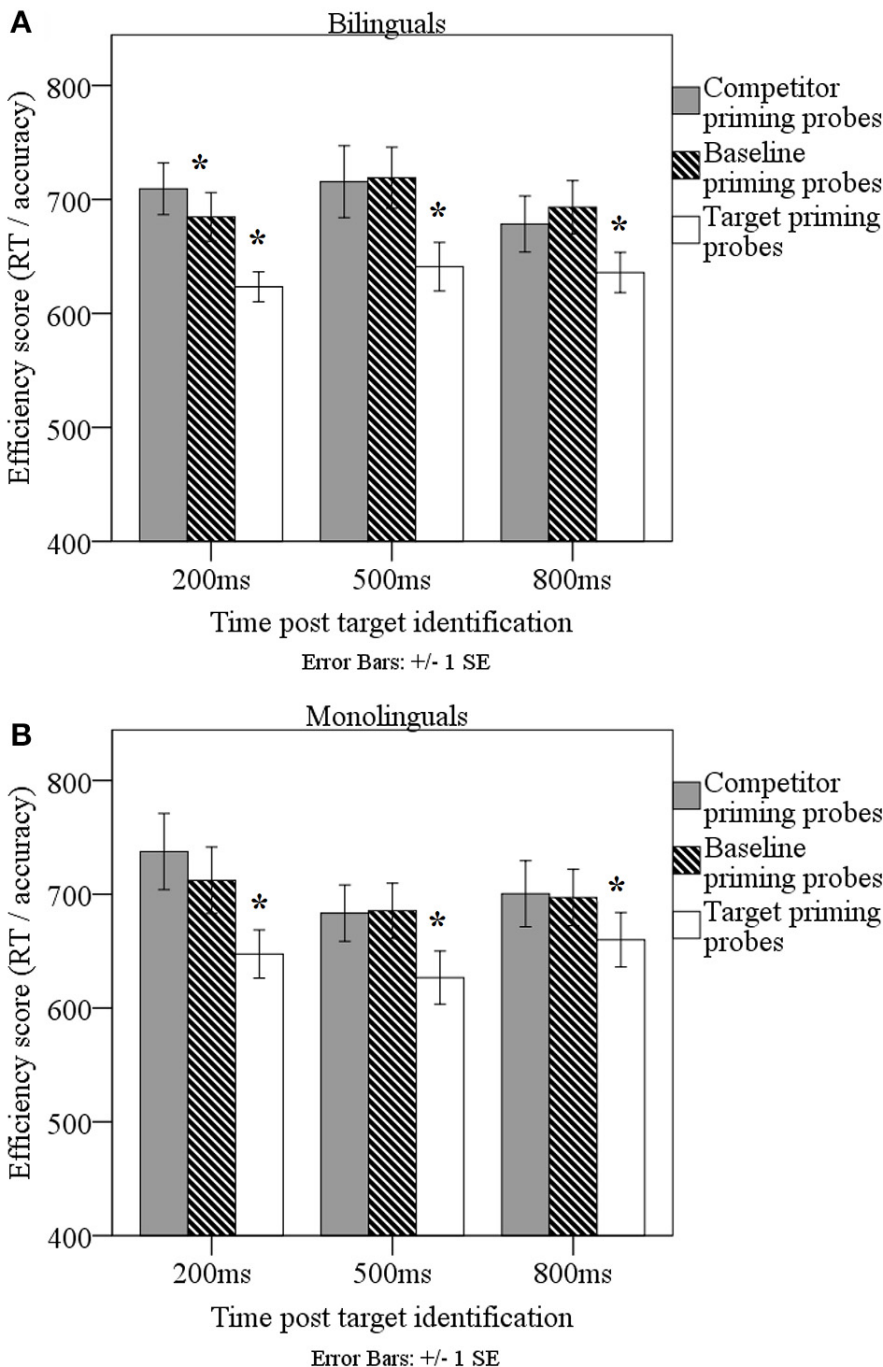
**Responses as efficiency scores (ms/proportion correct) to competitor priming probes (gray) and target priming probes (white), relative to baseline priming probes (striped), in bilinguals (A) relative to monolinguals (B)**. ^*^p < 0.01.

To examine residual inhibition of competitor symbols after target identification, a mixed linear model was employed with fixed effects including priming probes placed in previous baseline and competitor locations (baseline, competitor; baseline: baseline) and inter-stimulus interval (200, 500, 800; baseline: 200), as well as language group (bilingual, monolingual; baseline: monolingual). In addition, z-transformed PPVT scores and post-training accuracies were separately entered as continuous variables. Finally, participants were entered as random effects on the slopes of both competitor and inter-stimulus interval effects. Results yielded an interaction between competitor location and inter-stimulus interval: At 200 ms post-target identification, response efficiency was significantly longer and less accurate to competitor probes (*M* = 722 ms/proportion correct, *SE* = 16.9) than to baseline probes (*M* = 698.7, *SE* = 16.9), *b* = −38.6, *SE* = 15.9, *p* < 0.05. This finding suggested residual inhibition of the competitor at 200 ms post-target identification. While the three-way interaction between language group, inter-stimulus interval, and priming probe did not reach significance, *p* > 0.1, planned follow-up contrasts suggested that the difference between competitor and baseline probes was significant for bilinguals, *t*_(26)_ = −2.6, *p* < 0.05, but not monolinguals, *t*_(26)_ = −1.9, *p* = 0.07. This finding suggests that, while both bilinguals and monolinguals showed patterns of residual inhibition at 200 ms post-target identification, this effect was somewhat more robust in bilinguals, see Figure 7.

In addition, an interaction between language group and PPVT performance was again present, *b* = −114.6, *SE* = 35.4, *p* < 0.01: Monolinguals with higher PPVT scores responded to priming probes with greater efficiency (*R*^2^ = 0.227), while no such effect was present in the bilinguals (*R*^2^ < 0.001). Consideration of Spanish vocabulary in bilinguals did not change this pattern (combined PPVT and TVIP: *R*^2^ for bilinguals = 0.004; TVIP only: *R*^2^ for bilinguals = 0.016). Finally, when learning success was entered into the model instead of vocabulary knowledge, no effect involving learning success reached significance. Together, these findings suggest that (1) a pattern of residual inhibition was identified at 200 ms post-target identification but not at 500 or 800 ms, (2), these inhibition effects were particularly robust in bilinguals, and (3) neither receptive vocabulary knowledge nor symbol learning success modulated these inhibition effects.

#### Residual facilitation of target symbols

To examine residual facilitation of target symbols after target identification, a mixed linear model was employed with fixed effects including priming probes placed in previous baseline and target locations (baseline, target; baseline: baseline) and inter-stimulus interval (200, 500, 800; baseline: 200), as well as language group (bilingual, monolingual; baseline: monolingual). In addition, z-transformed PPVT scores and post-training accuracies were separately entered as continuous variables. Finally, participants were entered as random effects on the slopes of both competitor and inter-stimulus interval effects. Findings yielded a main effect of priming probe location, with shorter and more accurate responses on target (*M* = 642.4 ms/proportion correct, *SE* = 14.2) than baseline probes (*M* = 700.2 ms/proportion correct, *SE* = 14.2), *b* = −54.4, *SE* = 15.0, *p* < 0.001. When PPVT performance was entered into the model, we again found the previously described interaction between language group and PPVT, *b* = −104.8, *SE* = 33.6, *p* < 0.01, *R*^2^_bilinguals_ < 0.001; *R*^2^_monolinguals_ = 0.226. In bilinguals, inclusion of the combined PPVT/TVIP score (*R*^2^ = 0.002) or the TVIP score (*R*^2^ = 0.014) did not alter this pattern. Finally, when learning success was entered into the model, no significant effects emerged involving training success. Together, these findings suggest robust residual target activation across language groups and inter-stimulus intervals.

#### Associations between residual and Stroop inhibition

Finally, we examined the relation between residual competitor inhibition after tone-to-symbol identification and performance on a nonlinguistic Stroop task. Stroop performance was analyzed with a mixed linear model with fixed factors including condition (center, congruent, incongruent; baseline: center) and language group (bilingual, monolingual; baseline: monolingual), and with participants entered as a random effect on the slope. Results yielded a main effect of condition, with responses on congruent trials (*M* = 458.0 ms/proportion correct, *SE* = 8.1) significantly faster and more accurate than responses on center trials (*M* = 482.9 ms/proportion correct, *SE* = 8.2), *b* = −24.7, *SE* = 9.0, *p* < 0.01, and with incongruent trials (*M* = 585.9 ms/proportion correct, *SE* = 8.1) significantly slower and less accurate than center trials, *b* = 111.0, *SE* = 9.0, *p* < 0.001. No other effects were significant, suggesting equivalent Stroop inhibition performance across the bilingual and monolingual groups.

Consistent with Blumenfeld and Marian (2011), negative priming effects were compared with the difference score between congruent and incongruent Stroop reaction times, with smaller effects reflecting better abilities to ignore location information. The z-transformed Stroop effect was entered as a continuous variable into the previously-presented mixed linear model examining priming probes. Since relations between residual inhibition and Stroop performance were of interest, the dependent variable in this model was the negative priming effect (baseline probes minus competitor probes). A three-way interaction emerged between language group, inter-stimulus interval, and Stroop performance: at 500 ms post-target identification, in bilinguals, a smaller Stroop effect was associated with less residual competitor inhibition, relative to monolinguals, *b* = 38.9, *SE* = 18.8, *p* < 0.05, *R*^2^_bilinguals_ = 0.14, *R*^2^_monolinguals_ = 0.02.

## Discussion

In the current study, we compared bilinguals' and monolinguals' performance during a nonlinguistic learning task that involved mapping tones to symbols within a novel symbolic system that consisted of three distinctive features (timbre, pitch, and duration). Both learning and subsequent processing were examined in bilinguals vs. monolinguals. Subtle differences were evident across the two groups, particularly in the processing domain. These findings suggest that, even when bilingual advantages are not present, bilinguals may differ from monolinguals on tasks that resemble lexical mapping and involve competition resolution.

### Acquisition of novel tone-to-symbol mappings in bilinguals vs. monolinguals

On the sound-to-symbol matching task, bilinguals did not show a learning advantage. These results are consistent with previous findings that bilingual learning advantages may be determined by how the novel information relates conceptually to the previously established language systems. For example, Kaushanskaya and Rechtzigel (2012) suggest that learning of a novel word that is tied to a concrete (vs. abstract) translation equivalent will more widely activate bilinguals' previous two languages and may thus yield a more facilitative context for learning and integration of new knowledge. In contrast to Kaushanskaya and Rechtzigel's concrete learning condition, the current study required participants to map a new symbolic system in the absence of relevant previous conceptual representations. In this sense, the current study may be likened to an extreme version of Kaushanskaya and Rechtzigel's abstract word learning condition, and is consistent with the prediction that bilinguals may only outperform monolingual learners if their previous knowledge can be directly employed to scaffold new learning.

Perhaps because our task does not relate to bilinguals' previous language knowledge, the current findings stand in contrast with a previous nonverbal learning task where bilinguals showed advantages in learning a Morse code system (Bartolotti et al., 2011). One possible explanation for the absence in auditory processing advantages in the present study is that the current bilinguals had no previous language-based experience with the pitch, timbre, and duration dimensions in the current study, and in fact performed equivalently to monolinguals when learning these dimensions. Indeed, previous research has suggested that bilingual experience may reconfigure attention to linguistic and extralinguistic cues in the environment based on their relevance in previous learning experiences (e.g., Deutsch et al., 2004; Bialystok et al., 2005; Brojde et al., 2012). While natural language processing relies on listeners' capacity to make distinctions between pitch, timbre (i.e., formant structure) and duration, it is possible that the nature and constellation of these dimensions was too far removed from English-Spanish processing to allow for transfer of skills. For example, it is possible that Spanish-English bilinguals have some previous training on the fine-grained duration dimension that was critical in Bartolotti et al.'s learning task, given their awareness of temporal phonetic characteristics across their two languages, such as voice onset time discrimination in English vs. Spanish (e.g., Ju and Luce, 2004). However, the challenging combination of pitch, timbre, and duration dimensions may have differed from their previous linguistic experiences and clouded potential subtle advantages (e.g., Krumanshl and Iverson, 1992).

It is also possible that subtle cognitive differences between bilinguals and monolinguals contributed to advantages on Bartolotti et al.'s learning task but not the current learning task. For example, bilingualism has previously been associated with advantages in auditory working memory (e.g., Adesope et al., 2010), and such advantages in working memory may in turn be linked to statistical learning success (Misyak and Christiansen, 2012). While Bartolotti et al. did not include an auditory working memory task (only a forward digit span task was included), bilinguals in the current study did not differ from their monolingual peers on an auditory backward digit span measure. Yet, auditory working memory skills may in part account for bilinguals who were not successful learners. Specifically, across all linguistic and cognitive measures, follow-up analyses did not yield significant differences between unsuccessful (<65% accuracy) and successful (>65% accuracy) bilingual learners, with only a marginal difference in numbers reversed present between the two groups (*p* = 0.076). While no significant correlation was present between post-training accuracy and numbers reversed in the bilingual non-learners (*r* = 0.33, *p* > 0.1), this working memory difference nevertheless may have contributed to learning outcomes. In fact, a positive correlation between overall accuracy and numbers reversed skills was present across all bilingual learners and non-learners (*r* = 0.4, *p* = 0.01). It is thus possible that reliance on working memory within the bilingual group was in part responsible for the larger proportion of weaker bilingual learners. Working memory has previously been linked to learning outcomes (e.g., Papagno and Vallar, 1995), and lower working memory in the current bilingual sample was also associated with lower PPVT scores (*r* = 0.4, *p* < 0.05, for similar findings, see Kaushanskaya et al., 2011). No such correlations were found in monolinguals (working memory and training outcomes: *r* = 0.04, *p* > 0.5; working memory and vocabulary: *r* = 0.17, *p* > 0.4).

Beyond auditory working memory, several factors may account for the low tone-to-symbol mapping accuracies in the bilingual non-learners. In fact, combined learning of novel auditory and visual information was challenging for most participants (see post-training accuracies). For example, the length of both the training and processing phases required sustained attention and thus motivation to perform well. It may therefore be that sustained attention skills in general differentiated learners from non-learners, a possibility that can be explored in future research. Interestingly, while Spanish skills and language immersion were not significantly related to learning outcomes in the successful bilingual learners, in the bilingual non-learners higher Spanish skills and less English exposure were related to more successful learning (TVIP: *r* = 0.68, *p* < 0.05; English exposure: *r* = −0.83, *p* < 0.01). Of the non-learners, 9 were Spanish-English bilinguals, one was a simultaneous bilingual, and one was an early English-Spanish bilingual. Thus, in the bilinguals who struggled to learn, skill in the native language appeared to support tone-to-symbol mapping. The reason for this observed link between L1 vocabulary and learning performance in monolinguals and bilingual non-learners may be that underlying cognitive strengths facilitate both vocabulary acquisition and better task performance. It is possible that L1 vocabulary is a particularly strong indicator of underlying word learning skills while L2 vocabulary may be more context-specific and thus a reflector of experience more than word-learning skills *per se*. In sum, the current findings contribute to limiting the scope of bilingual learning advantages. Further they raise new questions on the nature of bilingual learning advantages, as well as pre-requisite cognitive-linguistic skills, perhaps suggesting that aspects of bilingualism may provide richer opportunities for scaffolding during specific new learning contexts, but that bilingual experience may not modulate fundamental learning mechanisms.

### Sound-to-symbol processing differences between bilinguals and monolinguals

Studying processing of newly-learned nonlinguistic information can eliminate group differences in content knowledge associated with bilingual status, thus providing an opportunity to compare competition resolution across groups in the absence of proficiency effects. Current findings across bilinguals and monolinguals that were equivalent on learning outcomes suggest subtle processing differences between the two groups. These differences emerged only when we examined the relation between sound-to-symbol retrieval and previous vocabulary knowledge and when the time course of inhibitory control was considered.

During the processing task, the bilinguals and monolinguals, who had attained similar skill levels with the new symbol system, also showed similar symbol retrieval efficiency. Moreover, consistent with previous explanations of bilingual retrieval disadvantages (e.g., Ivanova and Costa, 2008; Gollan et al., 2011b), participants who had attained lower learning outcomes on the novel tone-to-symbol system also showed less efficient retrieval skills. This relation between learning success and subsequent retrieval efficiency was present to an equal extent in both bilinguals and monolinguals, mimicking previous patterns from linguistic tasks (Gollan et al., 2008; Whitford and Titone, 2012), and confirming that less robust learning of content influences sound-to-content links and shapes retrieval success.

While retrieval efficiency could be in part explained by previous learning success in both bilinguals and monolinguals, a stronger link was identified between previous vocabulary knowledge and tone-to-symbol retrieval in the monolingual group. Specifically, in monolinguals, learners who had stronger English receptive vocabulary skills (as indexed by the PPVT) also were more efficient in retrieving sound-to-symbol mappings in the processing environment where these items had to be identified from competing alternatives. This association between receptive vocabulary knowledge and retrieval efficiency was not limited to trials with competitor items, but was found across competitor *and* no-competitor trials. These findings suggest that competition resolution during tone-to-symbol mapping was not modulated by previous receptive vocabulary. Rather, it appears that the ability to efficiently identify a newly-learned sound-to-symbol mapping among four alternatives was positively influenced by previous vocabulary. It is thus possible that skills that aid in the mapping of new vocabulary transferred to the novel task. Further, this effect also persisted during monolinguals' priming trials, perhaps suggesting that higher-vocabulary monolinguals used fewer cognitive resources during sound-to-symbol trials, allowing quicker responses on priming probes, or that higher-vocabulary monolinguals deployed attentional processes more efficiently in orienting toward relevant information on the displays.

In contrast to monolinguals, no association was found between bilinguals' English receptive vocabulary and their performance on the sound-to-symbol mapping or priming trials. When combined English/Spanish or Spanish-only receptive vocabulary skills were considered, this association was not significantly strengthened. It is possible that, in monolinguals, a more centralized and less distributed lexical system may better capture general word learning skills and related cognitive factors that might contribute to mapping a new symbolic system. It is possible that since, in bilinguals, vocabulary skills are frequently more context-specific due to language immersion tied to specific social settings, it is more challenging to index their core vocabulary knowledge through standardized measures such as the ones employed here. As a result, core knowledge that may point to underlying word learning skills was perhaps not as successfully indexed in the bilinguals. Alternatively, it is possible that bilinguals, due to word-learning experiences across linguistic contexts, may have word mapping skills that are not necessarily associated with their overall word knowledge. Interestingly, in the bilinguals who did not succeed on the learning task, a link between Spanish receptive vocabulary and sound-to-symbol mapping success *was* in fact evident. These findings must be treated with care given the small sample of bilingual non-learners (*n* = 11). Yet, they speak to a shared scaffolding mechanism for newly learned sound-to-symbol mappings in monolinguals and bilinguals. Additional research is needed to examine L1 and L2 lexical contributions to novel word learning in bilinguals. In sum, the ability to identify tone-to-symbol targets among competing options was modulated by different yet related variables in bilinguals and monolinguals, with learning success predicting retrieval efficiency in both groups, but with previous vocabulary knowledge predicting symbol retrieval efficiency more in monolinguals than bilinguals.

In addition to similarities in retrieval skills, monolinguals and bilinguals also showed similar competition effects within the novel symbol system. Findings of similar competition effects in monolinguals and bilinguals are consistent with previous language studies where linguistic competition resolution was examined and similar competition resolution patterns were found in the two groups (e.g., for lexical competition during word recognition, see Blumenfeld and Marian, 2011; for competition within a sentence context, see Paap and Yunyun, 2014). In the linguistic domain, comparisons of competition effects in bilinguals vs. monolinguals may be influenced by group differences in experience and proficiency, potentially obscuring bilingual advantages in competition resolution. However, the current findings suggest that, based on equivalent training and attainment, competition effects prior to target identification continue to have the same magnitude in the two groups.

Further, competition effects were not modulated by previous vocabulary knowledge or by learning success. These results are consistent with previous findings that language-based competition effects may not be modulated by proficiency during naming (e.g., see Marian et al., 2013, for equivalent Stroop effects across trilinguals' languages with varying proficiency levels). Similarly, Marian and Spivey (2003) found comparable lexical competition effects in L1 and L2 during auditory word identification in proficient late bilinguals. Nevertheless, other sources suggest that, in bilinguals, conflict monitoring skills may be honed to better identify ambiguities as they arise (e.g., Abutalebi et al., 2012). It is possible that novel representations must be more established before effects of previous experience on competition resolution can become visible. As might be expected in very novice learners who might show more variability in responses (e.g., Hulstijn et al., 2009), considerable variability across items and participants may have occluded subtle influences on competition effects at this early stage of learning. As such, a possible relation between competition resolution and previous vocabulary can be examined at various proficiency levels in future research.

While competition effects were similar in bilinguals and monolinguals, subtle group differences emerged in the nature of inhibition mechanisms that were deployed to resolve this competition. At 200 ms post-target identification, significant competitor inhibition effects were identified for bilinguals, with somewhat less robust inhibition effects in monolinguals. Interestingly, smaller Stroop inhibition effects were associated with less residual competitor inhibition at 500 ms post-target identification for bilinguals, with no such correlations in monolinguals. This correlation is suggestive of a time window where inhibition may be gradually lifted, given the absence of significant inhibition effects at this time. Together, it appears that inhibition effects lingered somewhat longer in bilinguals. These findings are consistent with Treccani et al. (2009)'s findings on a nonlinguistic priming task, and suggest that bilinguals may exert somewhat stronger inhibition effects than monolinguals to separate competitors from targets in novel symbolic processing environments.

While in the nonlinguistic domain findings suggest that bilinguals may maintain inhibition longer than monolinguals, a different pattern may be present in the linguistic domain. In a linguistic context that is analogous to the current study, Blumenfeld and Marian (2011) showed inhibition of linguistic competitors at 500 ms post-target identification for monolinguals but not bilinguals. Instead, a correlation was present where bilinguals with smaller Stroop effects showed less residual competitor inhibition at 500 ms post-target identification. Therefore, while the timecourse of inhibition appears identical across nonlinguistic and linguistic domains in bilinguals, monolinguals show more sustained inhibition effects in the linguistic domain. Additional research is needed to better explicate this difference across modalities. It is possible that, as bilinguals become more proficient with content knowledge (as is the case in the linguistic domain), they may show faster competition resolution, also leading to earlier release of inhibition mechanisms post-target identification (e.g., Mishra et al., 2012). Monolinguals may sustain inhibition longer in a linguistic environment to protect against intrusion from similar-sounding words, while bilinguals may release such inhibition sooner to allow for language switches. As was the case for competition resolution prior to target identification, the magnitude of residual inhibition effects post-target identification was not modulated by experiential factors. These findings suggest that, at least for newly-learned symbolic information, the magnitude of inhibition may not be related to linguistic knowledge *per se* but may relate to participants' domain-general cognitive skills.

Together, findings from the linguistic and nonlinguistic processing domains suggest that, while bilinguals and monolinguals are very similar in their efficiency of competition resolution (as indexed by response efficiency on tone-symbol mapping trials with vs. without competitors), they show subtle differences in the time course along which they maintain inhibition after word identification. In turn, as in Blumenfeld and Marian (2011), bilinguals and monolinguals showed equivalent magnitudes of target facilitation during the time immediately following target identification. It is likely that target facilitation acts as a competition resolution mechanism that complements inhibition of irrelevant information (e.g., Paradis, 2004), and previous work suggests that it outlasts inhibition effects across time (Tanaka and Shimojo, 2000). The current findings where residual activation was probed at three times post-target identification, confirm these patterns.

## Conclusions

The current task was developed to mimic processes of word learning and auditory word recognition in bilinguals vs. monolinguals, while putting the two groups on equal footing in terms of experience and skill with the underlying information. Findings suggest that retrieval and competition in the nonlinguistic domain were comparable to previous findings in the linguistic domain (e.g., Marian and Spivey, 2003; Blumenfeld and Marian, 2011; Gollan et al., 2011b) and allowed us to isolate potential roles of previous experience on domain-general skills, free of experiential differences in content. Within this framework, we were able to examine learning and processing separately as well as in combination. Highly similar learning outcomes in bilinguals vs. monolinguals could be explained because the nature of the novel information was not linked to previous experience. When the newly-learned material was presented in combination with similar-sounding competitors, competition resolution mechanisms were engaged that showed subtle bilingual-monolingual differences that are likely related to previous bilingual experience.

In the current study, relying on pitch and timbre cues was not central to Spanish-English bilinguals' previous bilingual experiences and, perhaps as a result, no learning advantages were identified. Future work can further extend these findings by examining participants who have had previous learning experience with pitch perception, such as speakers of tone languages, or individuals who have previous experiences with both pitch and timbre perception, such as musicians (e.g., Chartrand and Belin, 2006; Kraus et al., 2009). Findings are consistent with previous results that bilingual experience influences the nature of competition resolution during processing. However, the current findings show only subtle differences between bilinguals and monolinguals instead of general bilingual advantages in competition resolution. Therefore, the current set of results is most consistent with the prediction that bilingual advantages, if they emerge, are specific and to be identified in areas that have direct overlap with previous experience.

### Conflict of interest statement

The authors declare that the research was conducted in the absence of any commercial or financial relationships that could be construed as a potential conflict of interest.
