# Supplementary material for: Learning and processing of nonverbal symbolic information in bilinguals and monolinguals
Source: Front Psychol. 2014 Oct 16;5:1147. doi: 10.3389/fpsyg.2014.01147 (PMC4199272; doi:10.3389/fpsyg.2014.01147)
Supplement: Supplementary file 1 [file DataSheet1.DOCX]

Appendix A. Descriptions of six sample tone-to-symbol correspondences that were presented to participants during the training session, together with corresponding descriptions of the relation between tones and symbols.

| **Visual symbol presented to participants while they heard the corresponding tone.** | **Text presented to participants** |
| --- | --- |
|  | **This tone represents a sine wave (or less piercing) tone because it is black, a high pitch tone because the bar is located at the top of the vertical line, and a long tone because the bar is long. Therefore this tone-picture combination is called *high long black*.** |
|  | **This tone represents a sine wave (or less piercing) tone because it is black, a high pitch tone because the bar is located at the top of the vertical line, and a short tone because the bar is short. Therefore this tone-picture combination is called *high short black*.** |
|  | **This tone represents a sine wave (or less piercing) tone because it is black, a mid pitch tone because the bar is located in the middle of the square, and a long tone because the bar is long. Therefore this tone-picture combination is called *mid long black*.** |
|  | **This tone represents a sine wave (or less piercing) tone because it is black, a mid pitch tone because the bar is located in the middle of the square, and a short tone because the bar is short. Therefore this tone-picture combination is called *mid short black*.** |
|  | **This tone represents a sine wave (or less piercing) tone because it is black, a low pitch tone because the bar is located at the bottom of the vertical line, and a long tone because the bar is long. Therefore this tone-picture combination is called *low long black*.** |
|  | **This tone represents a sine wave (or less piercing) tone because it is black, a low pitch tone because the bar is located at the bottom of the vertical line, and a short tone because the bar is short. Therefore this tone-picture combination is called *low short black*.** |
